# Strong but Fragmented Memory of a Stressful Episode

**DOI:** 10.1523/ENEURO.0178-23.2023

**Published:** 2023-09-01

**Authors:** Anna-Maria Grob, Denise Ehlers, Lars Schwabe

**Affiliations:** Department of Cognitive Psychology, Institute of Psychology, Universität Hamburg, Hamburg 20146, Germany

**Keywords:** fNIRS, memory, recognition priming, stress

## Abstract

While it is commonly assumed that stressful events are vividly remembered, it remains largely unknown whether all aspects of memory for a stressful episode are enhanced. In this preregistered study, we tested whether stress enhances later remembering of individual elements of a stressful episode at the cost of impaired processing of the association between these elements. Therefore, male and female participants (*N* = 122) underwent a stressful (or control) episode during which they encoded a series of stimuli. To investigate stress effects on the memory for individual events and the links between these, we used temporal sequence effects in recognition memory tested 24 h after encoding. Specifically, we tested whether stress would affect the memory enhancement for a target item if this is preceded by another item that also preceded the target during encoding (recognition priming). Our results showed that participants recalled single events encoded under stress better than those encoded under nonstressful conditions, but were less able to leverage the temporal sequence of events encoded under stress to cue memory at delayed recall, reflected in reduced memory for items preceded by the item that preceded them also during encoding. Functional near-infrared spectroscopy further revealed that encoding under stress was accompanied by opposite changes in inferotemporal and dorsolateral prefrontal areas. Together, our data suggest that acute stress induces a mode of memory formation that results in strong but less integrated memories.

## Significance Statement

Stress has a major impact on memory, with critical implications for stress-related mental disorders such as posttraumatic stress disorder (PTSD). For trauma memory in PTSD, however, there is a remarkable contradiction: while memory for the trauma is typically so strong that it leads to involuntary recall, trauma memory is often fragmented and disintegrated. We hypothesized that these memory distortions might be because of a mode of memory formation under stress that enhances memory for individual events but impairs the processing of associations between these. In line with our hypothesis, we found that stress resulted in strong but fragmented memories. Stronger but less integrated memory was accompanied by specific changes in frontal and temporal brain areas.

## Introduction

Stressful events have a significant impact on our memory (Diamond et al., 2007; Luksys and Sandi, 2011; Schwabe et al., 2022). In particular, stressful events are typically much better remembered than mundane events (Sandi et al., 1997; Vogel and Schwabe, 2016b; Kalbe et al., 2020). This memory enhancement for stressful events is attributed to the action of noradrenaline and glucocorticoids on prefrontal and medial temporal lobe areas, including the hippocampus (Kim and Diamond, 2002; Roozendaal et al., 2009). In addition to modulating single brain areas, these stress mediators induce a reconfiguration of large scale neural networks, from an executive control network toward a salience network that prioritizes emotionally salient information (Hermans et al., 2011, 2014). Although being generally highly adaptive, the memory enhancement for stressful events can become maladaptive and contribute to the painful memory for traumatic events that is a hallmark of posttraumatic stress disorder (PTSD; Pitman et al., 2012).

For trauma memory in PTSD, there is, however, a remarkable contradiction: although the memory for the trauma is exceptionally strong, giving rise to involuntary intrusions even years after the traumatic event, trauma memory is often fragmented and disintegrated in PTSD (Brewin, 2011; Bisby et al., 2020). How can these seemingly contradictory observations be reconciled? A potential explanation relates to the stress-induced reconfiguration of large-scale neural networks and the closely linked shift from prefrontal and hippocampal to dorsal striatal control of memory under stress (Schwabe, 2017). While the prefrontal cortex (PFC) and hippocampus are crucial for the processing of associations and the mnemonic integration of events (Murray and Ranganath, 2007; Shohamy and Wagner, 2008), the dorsal striatum processes individual stimuli but less the association between these stimuli (Packard and Knowlton, 2002). In line with this idea of a stress-induced shift from associative memory to stimulus-based memory, stress or glucocorticoids before encoding has been shown to reduce the incorporation of contextual details into a memory trace (Schwabe et al., 2009; van Ast et al., 2013; Sep et al., 2019; Simon-Kutscher et al., 2019) and to exert differential effects on item and context memory (Goldfarb et al., 2019; Kamp et al., 2019; Antypa et al., 2022). Based on these findings, we hypothesized that acute stress may induce an altered memory formation mode characterized by enhanced processing of individual elements of an episode but impaired encoding of the links between these elements, thus resulting in strong but fragmented memories resembling trauma memory in PTSD.

In this preregistered experiment, we tested this hypothesized mode of memory formation under stress, defined as a threat to the individual’s homeostasis (McEwen, 2000), and the mechanisms involved herein. To investigate stress effects on the memory for individual items and the links between these, we leveraged temporal sequence effects in recognition memory. Specifically, the recognition of an event is significantly better when it is preceded by an event that occurred before it during encoding (Schwartz et al., 2005). This “recognition priming” effect implies that an association between events was formed during encoding which can then serve as a cue for subsequent recognition. Previous research showed that recognition priming is related to whether events are segmented or grouped in memory (DuBrow and Davachi, 2013; Rouhani et al., 2020) as well as to intrusions in PTSD (Michael and Ehlers, 2007). If stress enhances memory for individual events but impairs the mnemonic integration of these events, then stress should result in enhanced memory for isolated events but reduced recognition priming. Because stress is assumed to enhance memory only for the stressor itself and information directly related to it (Schwabe and Wolf, 2010; Kalbe et al., 2020), we developed a new stress protocol that included key elements essential for successful stress induction (Dickerson and Kemeny, 2004), while being directly related to the ongoing encoding task. Because the stress-induced shift toward the salience network is mediated primarily by noradrenaline (Hermans et al., 2011) and the delayed cortisol response may be too late to affect integrative encoding under stress, we predicted that enhanced item memory and impaired integrative encoding (as reflected in recognition priming) should be directly linked to autonomic activity. Moreover, to investigate the involved brain mechanisms, we employed functional near infrared spectroscopy (fNIRS). We focused on the dorsolateral prefrontal cortex (dlPFC) and inferior temporal gyrus (ITG), which are thought to be differentially modulated by stress (Hermans et al., 2014) and to play an important role in relational encoding and memory formation under stress (Murray and Ranganath, 2007; Henckens et al., 2009; Kalbe et al., 2020). We predicted that changes in these areas would be linked to both the enhanced memory for individual events and the reduced integration of events under stress. Further, we tested mediation models in which stress effects on item and integrative memory are (1) differentially mediated by increases in autonomic arousal and (2) mediated by changes in ITG and dlPFC activity, respectively.

## Materials and Methods

### Preregistration

This study was preregistered before the start of data collection at the Open Science Framework (OSF; https://osf.io/km9qs).

### Participants and experimental design

One hundred and twenty-six healthy volunteers between 18 and 35 years of age participated in this experiment. Exclusion criteria for participation were checked in a standardized screening interview and comprised any current illness or intake of nonprescription medication, life-time history of any mental or neurologic disorders, smoking, drug abuse, and pregnancy or lactation. Furthermore, women were not tested during their menses and excluded from participation if they used hormonal contraceptives which are known to modulate the endocrine stress response (Kirschbaum et al., 1999). The sample size was based on an a priori power calculation using GPOWER (Faul et al., 2007), which indicated that a sample of 119 participants is required to detect a medium-sized effect, as observed in previous studies of our lab on stress effects on related memory processes (Schmidt et al., 2013; Kluen et al., 2017; Kalbe et al., 2020), for the interaction between the between-subjects factor group (stress vs control) and the within-subject factor block (block 1 vs block 2) with a power of 0.95. We tested 126 participants because we expected a drop-out rate of ∼5%. Four participants had to be excluded from the analyses because of not showing-up for the second experimental day (*n* = 1), technical failure (*n* = 1) or lack of compliance with the instructions (*n* = 2), leaving a sample of 122 participants. All participants provided written informed consent before participation and received a compensation of €40 for participation. The study procedure was approved by the ethics committee of the Faculty of Psychology and Human Movement Sciences at the University of Hamburg (2020_319).

In a between-subjects design, participants were pseudo-randomly assigned to a stress [32 men, 28 women; age: mean (M) = 26.25 years, SD = 4.88 years] or control group (30 men, 32 women; age: M = 25.06 years, SD = 4.19 years) to ensure a comparable distribution of men and women across groups.

### Experimental procedure and materials

In order to control for the diurnal rhythm of major stress response systems, all testing took place in the afternoon between 12.30 pm and 6 pm.

#### Stimuli

The stimulus material consisted of 540 coloured pictures of outdoor scenes, obtained from open Internet platforms. For each of the participants, 360 of the 540 pictures were randomly selected for presentation during encoding, while the remaining 180 pictures were used as lures in the recognition test.

#### Day 1: encoding and stress manipulation

After participants’ arrival at the lab, they completed the State-Trait Anxiety Inventory (STAI; (Spielberger et al., 1983), Beck Depression Inventory (BDI; (Beck and Steer, 1987), and the Trier Inventory of Chronic Stress (TICS; Schulz et al., 2004), to control for potential group differences in state or trait anxiety, depressive mood, and chronic stress levels. Thereafter, baseline measurements of subjective stress ratings and salivary cortisol (see below) were taken, before participants were prepared for the peripheral physiological and fNIRS measurement. Next, we placed an electrode for electrodermal stimulation at participants’ lower leg and individually adjusted the shock intensity in a step-wise procedure to be highly unpleasant but not painful for the participant. Notably, this shock intensity adjustment procedure was performed both in the stress and control groups to rule out that this procedure could result in baseline encoding differences between groups. The shock electrode was removed again before the start of the encoding task.

**Figure 1.**
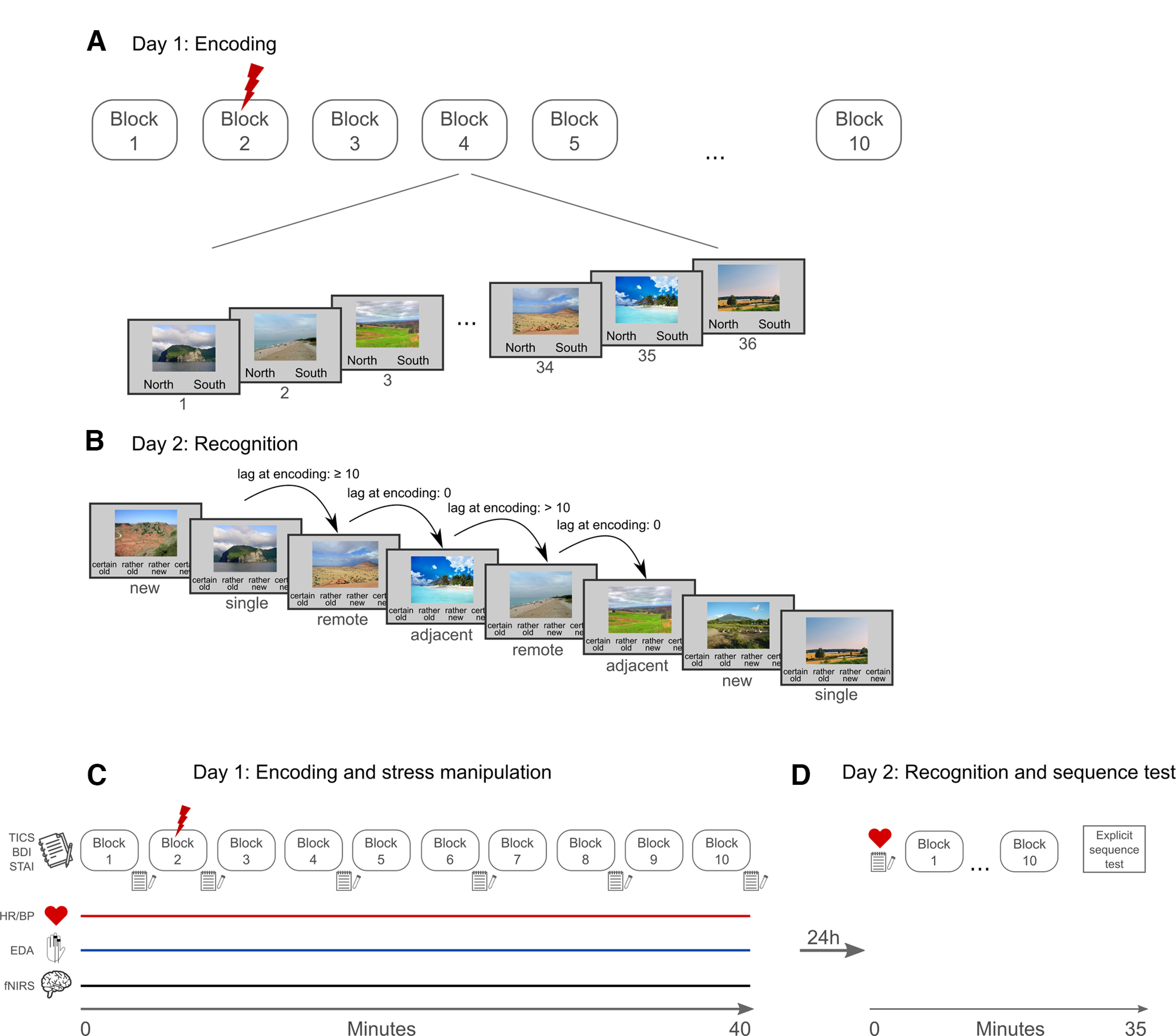
Overview of the experimental paradigm. ***A***, On the first experimental day, participants encoded a series of scene images distributed over 10 blocks, with 36 images each. For each picture, participants indicated whether the depicted scene is located on the northern or southern hemisphere. Importantly, in the second block, participants of the stress group received electric shocks that were purportedly depending on their response but in fact uncontrollable and further monitored by another experimenter and videotaped. During the entire encoding session, we measured cortical activity (using fNIRS) as well as autonomic arousal. ***B***, On the subsequent day, ∼24 h after encoding, participants completed a recognition test that allowed us to test for both the memory for individual items and recognition priming effects that are indicative for the extent to which associations between items had been encoded. Specifically, the recognition test contained (1) *single* items, which were presented after a new item and their memory could accordingly not be cued by the preceding stimulus; (2) *remote* items, which were preceded by another old item that was, however, presented with a lag of at least 10 items from the target item during encoding and for which there should be not or only small recognition priming effects; and (3) *adjacent* items, which were preceded by an item that immediately preceded this target also during encoding, thus enabling this items to cue memory for the target item. The memory for adjacent items indicates to what extent the association between items was encoded. ***C***, The encoding session on day 1 took ∼40 min (10 blocks à 144 s, 1–2 min between blocks) and we measured heart rate (HR), blood pressure (BP), electrodermal activity (EDA) as well as cortical activity using fNIRS throughout the task. Moreover, we took subjective stress ratings and saliva samples repeatedly across the task. Further, we measured chronic stress (TICS), depressive mood (BDI), and anxiety levels (STAI) before the encoding task. ***D***, On day 2, we took again heart rate and blood pressure measurements, another saliva sample and stress rating before the recognition test started that was key to the present study. In addition, participants performed an explicit sequence memory task after recognition testing (see Fig. 2).

The encoding task consisted of 10 blocks (Fig. 1). We conceptualized these blocks as episodes within which items should be bound together and assumed that this binding is also relevant for the later cueing by other items in the recognition test (i.e., recognition priming). In each of these blocks, participants saw 36 pictures of outdoor scenes. Each picture was presented in the center of a computer screen for 4 s and participants were instructed to judge whether the outdoor scene depicted is located in the northern or southern hemisphere (no shocks were mentioned at this stage). The two response options (north vs south) were presented below the pictures and participants responded via right or left button press. Participants did not receive feedback about the correct answer. The specific response of the participant was not of interest in the context of this study but the rating was (1) supposed to promote sufficient encoding of the stimuli and (2) used for the subsequent stress manipulation. The first block of encoding served as baseline block and was identical for the stress and control groups.

The critical experimental manipulation took place in the second block of the encoding task. Before the start of this second encoding block, participants in the stress group were informed that they would now receive electric shocks if they responded incorrectly to the question of whether the scene depicted was located on the northern or southern hemisphere, i.e., aversive outcomes were purportedly dependent on responses to the encoded stimuli, thus establishing a link between the stress manipulation and the encoding task. Therefore, the shock electrode was again placed on the right lower leg in the stress group (but not in the control group). During the second encoding block, all participants of the stress group received 15 200 ms-shocks of individually determined intensity. These shocks were administered 2.5–3 s after stimulus onset (jitter 500 ms) and actually independent of the correctness of the participants’ responses and therefore uncontrollable. In addition, during this second block, participants in the stress group (but not those in the control group) were videotaped and critically evaluated by a second experimenter dressed in a white lab coat standing directly behind the participant. Participants were informed that this second experimenter is trained in behavioural analysis and would now evaluate their performance. This second experimenter entered the room only before the start of the second block and left the room immediately after the completion of the second block. This stress protocol thus combined several elements known to elicit robust stress responses, in particular threat of shock and social evaluation (Dickerson and Kemeny, 2004; Robinson et al., 2013), and was purportedly directly related to the encoding task. In general, this stress manipulation was based on the idea that stress is induced by the combination of challenge and social evaluation (Dickerson and Kemeny, 2004), and was intended to model moderate stressors that occur repeatedly in everyday life. We restricted the stress manipulation to one encoding block of ∼2.5 min because (1) stress responses are known to be highly dynamic and we aimed to reduce the risk of, for instance, habituation effects which would have complicated the interpretation of our findings; (2) this duration is comparable to the duration of established stress protocols, such as the socially evaluated cold pressor test (Schwabe et al., 2008); (3) ethical considerations require to keep the duration of a stress manipulation to the minimum duration that is needed for an expected effect. For the control group, this second encoding block was identical to the first (baseline) block and control participants did not receive any specific instructions before the beginning of the second block. Importantly, no camera or second experimenter was present and the shock electrode was not re-attached to the participants’ leg, so that they knew they could not receive any electric shocks.

Blocks 3–10 were again, for both groups, identical to the baseline block (after block 2, the shock electrode was removed in the stress group). These blocks served to assess potential aftereffects of the stress exposure, related, for instance, to the experience of uncontrollability over aversive events (Maier and Seligman, 1976; Wanke and Schwabe, 2020) and the delayed action of cortisol on memory. Between blocks there was an interval of up to 3 min, during which stress measures were taken. To assess the effective stress manipulation, we measured subjective stress ratings on a scale from 0 (“not at all stressed”) to 100 (“extremely stressed”) immediately before and after encoding block 2, after encoding blocks 4, 6, 8, and 10 as well as 15 min after the encoding task. Moreover, we continuously measured blood pressure, heart rate, and electrodermal activity throughout the encoding task. For EDA, two participants were classified as outliers (>3 SD above the mean) and removed from the respective analyses. At the time points of the subjective ratings, we collected also saliva samples using Salivette (Sarstedt) collection devices, from which we analyzed concentrations of the stress hormone cortisol using a luminescence assay (IBL) at the end of data collection. In retrospect, these analyses suggested that cortisol concentrations were overall extremely low, which may be because of the fact that testing was performed in the late afternoon, near the evening nadir of the diurnal cortisol rhythm. Importantly, for ∼40% of the samples the indicated cortisol concentrations were either close to the lower sensitivity threshold of the assay or not even detectable, which questions the reliability of these data. Therefore, and because we did not assume a critical role of the delayed cortisol response on the encoding processes that are at the heart of this study (as explicitly indicated in the preregistration), we decided to refrain from an inclusion of the cortisol data in the data analyses.

#### Day 2: recognition memory testing

On the following day, participants returned to the lab. Day 2 testing took place in a different room than encoding on day 1 to avoid context-dependent memory effects and a potential modulation thereof by stress. Because both sleep (Diekelmann and Born, 2010) and the amount of rehearsal (Karpicke and Roediger, 2008) are known to influence memory performance, participants first completed short questionnaires on their sleep quality and duration in the night between the experimental days as well as on the amount of rehearsal after the encoding task on day 1 (Kalbe et al., 2020). Moreover, participants completed again the state version of the STAI, indicated their subjective stress level and collected a saliva sample before the recognition test started, to control for potential group differences in stress levels at test.

**Figure 2.**
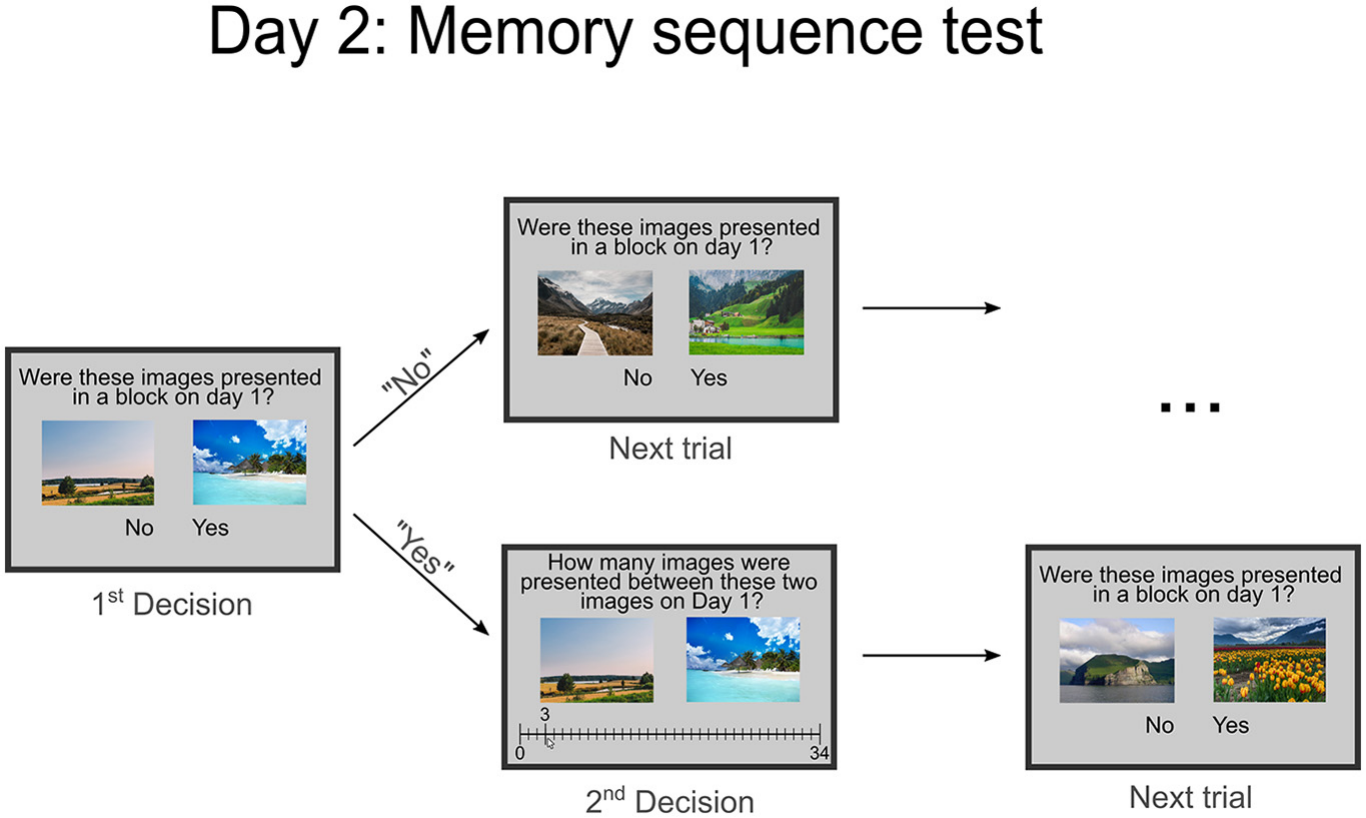
Explicit sequence memory test. At the end of experimental day 2, participants completed an explicit sequence memory test. In this test, participants saw two of the scenes that were encoded on day 1 and were asked to indicate whether these scenes were presented in the same encoding block or not. If participants indicated that these scenes were presented in the same block, they were then requested to indicate how many items were presented between these scenes in the respective encoding block (answers could vary between 0 and 34 items). The task was self-paced.

In the recognition test, participants saw the 360 pictures that were presented on days 1 and 180 new pictures (lures) sequentially on a computer screen (Fig. 2). For each of the pictures they were asked to indicate as quickly as possible (without time limit) on a scale from 1 to 4 (“certain old,” “rather old,” “rather new,” “certain new”) whether they had seen the picture on day 1 (“old”) or not (“new”). Critically, the trial sequence was predetermined allowing us to assess recognition priming effects depending on the block in which the stimuli were encoded on day 1. More specifically, unbeknownst to participants, the recognition test consisted also of 10 blocks (without any delay between blocks). Each of the 10 recognition blocks corresponded to one of the encoding blocks and comprised 54 trials in total: 18 lures, 12 single trials, 12 remote trials, and 12 adjacent trials. The definition of trial types was based on the item immediately preceding the target item in the recognition test. In single trials, an old item followed a new item, i.e., for these items there cannot be a recognition priming effect. In remote trials, an old item was presented after an item that was also old but was presented at a distance of ≥10 other items during encoding, i.e., there should be a minimal, if any, recognition priming effect for these items. In adjacent trials, however, an old item follows the item that preceded this item also during encoding. For these adjacent items, recognition priming effects should be largest (Schwartz et al., 2005). The sequence of trial types within a recognition block and the order of the blocks during recognition testing were completely randomized for every participant.

After the recognition test, participants completed a self-paced sequence test probing their explicit memory for the sequence of items during encoding. In this task, participants saw two old items presented side by side and were first requested to indicate by button press whether these items had been presented in the same encoding block on day 1. If they indicated that the items were not encoded in the same block, the next pair of images was presented. However, if participants responded that these two pictures were presented in the same encoding block, they were then asked to judge how many items were presented between these two items during encoding by selecting a response between 0 and 34 items (see Fig. 2).

### Peripheral physiological measurements and analysis

Indicators of autonomic arousal, blood pressure, heart rate, and EDA, were measured throughout the encoding task on day 1 using a BIOPAC MP160 system (BIOPAC Systems). Blood pressure and heart rate were continuously recorded with a finger cuff placed at the middle phalanx of the left index and middle fingers, using a noninvasive blood pressure amplifier (NIBP100D, BIOPAC Systems). Electrodermal activity was measured using a wireless BioNomadix system (BIOPAC Systems) with electrodes placed at the thenar and hypothenar eminences of the palm of the left hand. To ensure EDA responsivity, we asked all participants to take a deep breath at the beginning of the experiment, which is known to elicit an EDA response (Boucsein, 2012); the expected response was observed in all participants. Each of these peripheral parameters was averaged across the respective encoding block, which was assumed to lead to robust results. Nevertheless, we performed a baseline correction by using the 10-s periods before the start of an encoding block. Furthermore, we excluded participants with extreme data (i.e., >3 SD above the mean; see below).

### fNIRS recording and analysis

Cortical activation was measured with fNIRS throughout the encoding task on day 1. We used a NIRScout System (NIRx Medical technologies LLC, L.A., USA) with 16 sources and 16 detectors, forming 37 channels. The system was equipped with Avalanche Photodiodes ensuring an optimal signal-to-noise ratio and short-distance detectors that acquire extracerebral hemodynamic signals which are regressed out from cerebral signals and thus allowed us to control for blood pressure differences between treatment conditions. The used montage covered, in addition to sensory and motor control areas, in particular the dlPFC and ITG. Our focus on the ITG and dlPFC was primarily motivated by two lines of research. First, the ITG and dlPFC are two prominent cortical regions of the salience network (ITG) and the executive control network (dlPFC), respectively, and previous research indicated that stress is assumed to result in a shift from the executive control network to the salience network (Hermans et al., 2011, 2014; Schwabe et al., 2022). Hence, we predicted increased ITG and reduced dlPFC activity under stress. Second, the ITG and dlPFC have also been shown to be implicated in memory processes highly relevant for the present study. Specifically, the ITG has been linked to enhanced memory for stressful events (Henckens et al., 2009) and an earlier study from our lab showed that ITG activity was linked to memory for central features of a stressful episode (Kalbe et al., 2020). The dlPFC in turn has been related to more elaborate encoding processes, in particular relational memory encoding (Murray and Ranganath, 2007; Blumenfeld et al., 2011), which may be highly relevant for adjacent item memory.

Data were preprocessed in nirsLAB (v2016.01, MIRx Medical technologies LLC). We identified the detector saturation and interpolated consecutive channels if required. Data quality of the channels was inspected and if the CV was ≥15%, indicating a poor signal-to-noise-ratio, the channels were excluded. In an individual, first level analysis, we used prewhitening with autoregression and the 10 encoding blocks as regressors, all modelled with a hemodynamic response function. Our analyses focused on oxygenated hemoglobin and the contrast treatment block minus baseline block. The preprocessed fNIRS data were further processed using a MATLAB script that generated a matrix of the data across all channels and then integrated all channels that belonged to one topographical cluster, with each channel being weighted by the specificity of the channel for the respective brain region. The first level contrast was then taken to a second, group level focusing on the interaction of group × block. The exclusion of channels based on the CV criterion resulted in a signal loss in the cluster of interest for some of the participants, in particular for the ITG because optodes relevant for this area were particularly sensitive to head movement. The remaining sample sizes per region of interest were: dlPFC, 115 participants (62 control, 53 stress) and ITG, 64 participants (32 control, 34 stress).

### Statistical analysis

Subjective and physiological stress measurements were subjected to mixed-design ANOVAs with the between-subjects factor group (stress vs control) and the within-subject factor time point of measurement (10 encoding blocks). To assess the impact of stress on subsequent recognition priming, we analyzed hits, collapsed across “certain old” and “rather old” responses, by means of a mixed-design ANOVA with the between-subjects factor group (stress vs control) and the within-subject factors block (block 1 vs block 2; in additional analyses across all 10 encoding blocks or across the last eight blocks) and trial type (single vs remote vs adjacent). In an additional analysis, we tested also participants’ confidence in their memory by analyzing specifically their high confidence (“certain old”) hits. We further analyzed the false alarm rate with a *t* test, to test for potential group differences in response tendencies. Note that an analysis of the sensitivity index d’ or memory accuracy (hits minus false alarms) was not possible because there was no false alarm rate specifically for any of the trial types (single, remote, adjacent) but only an overall false alarm rate per block. Significant main or interaction effects were followed up by *post hoc* tests that were Bonferroni-corrected (*p*_corr_) if required. In case of violation of the sphericity assumption, Greenhouse-Geisser correction was applied. In addition to these ANOVA models, we analyzed correlations between memory parameters on the one hand and parameters of autonomic arousal and cortical activity on the other hand. We report the correlations both across the entire sample and separately for the stress and control groups. The correlation within groups is followed by a Fisher’s *z*-test to assess whether the correlations are reliably different from one another. Moreover, we performed regression analyses including the interaction term between the factor group (stress vs control) and the respective predictor. Only if this interaction term is significant, correlations within the separate groups can interpreted, whereas interpretation of the correlation across groups is allowed only if the interaction term is not significant. To further follow-up on these correlational analyses, we performed mediation analyses in which we tested whether stress effects on memory for single and adjacent items were mediated by changes in autonomic arousal (heart rate, blood pressure, or EDA) or cortical (i.e., ITG or dlPFC) activity. In these mediation analyses, we used group as predictor, single or adjacent item memory as outcome, and autonomic arousal measures or cortical activity as mediator. All reported p-values are two-tailed. All statistical analyses were performed with SPSS Version 25 and JASP version 0.16.4.

### Data and code availability

Data have been made publicly available and can be accessed at https://osf.io/s2z46/. All materials and scripts have been made publicly available and can be accessed at https://osf.io/s2z46/. This project has been formally preregistered before the start of data collection at https://osf.io/km9qs.

## Results

### Successful stress manipulation

**Figure 3.**
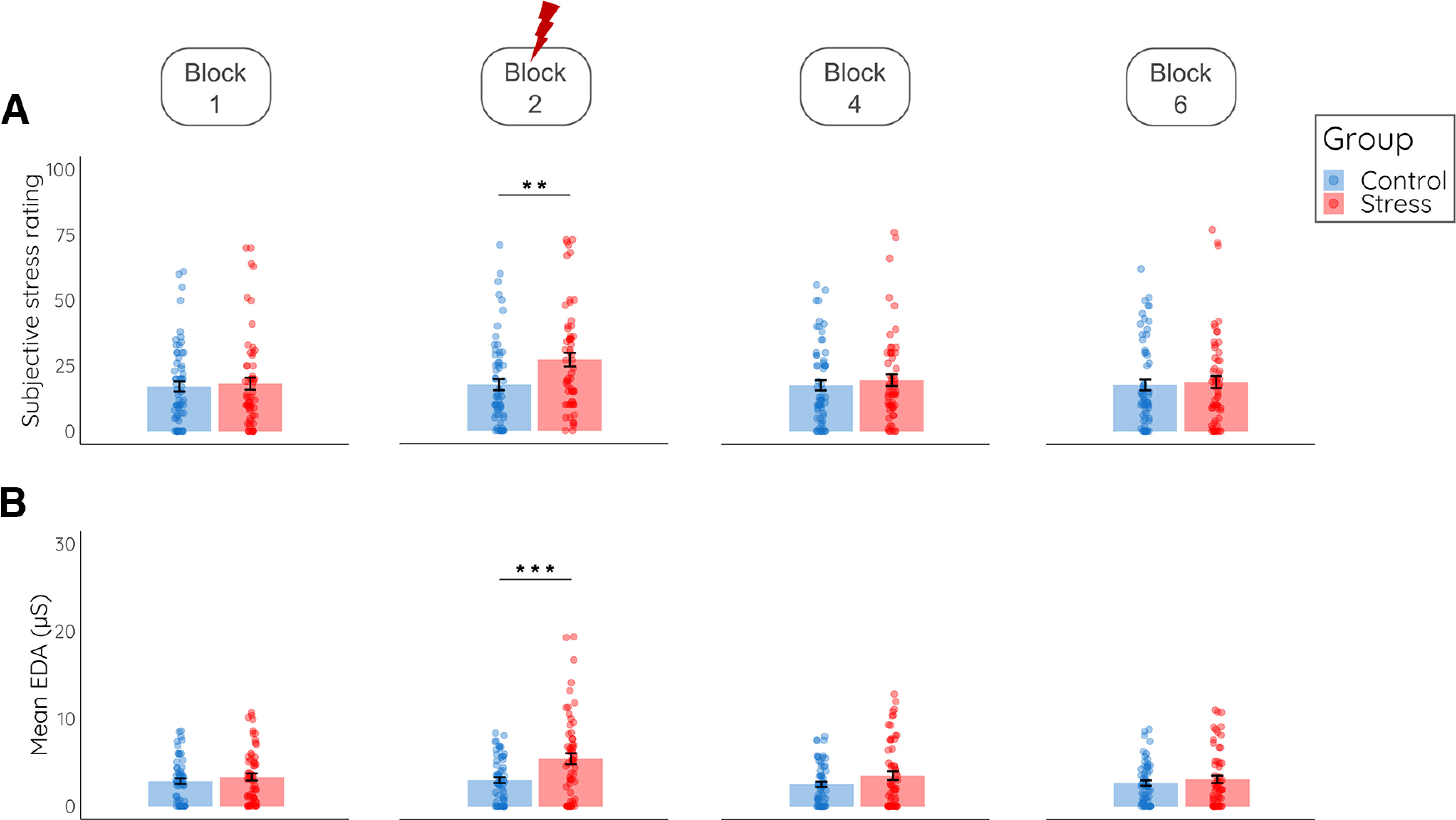
Subjective and physiological stress response. ***A***, Subjective stress ratings after the first, second, fourth and sixth block for the control and the stress group. ***B***, Mean electrodermal activity (EDA) during the first, second, fourth and sixth block. For EDA data across all blocks (see Table 1). Data represent means (±SE); **p < 0.01, ***p < 0.001.

Subjective and physiological stress measures confirmed the successful stress manipulation. For subjective stress ratings, there was a significant time × group interaction (*F*_(2.955,348.646)_ = 5.72, *p *=* *0.001, η_p_^2^ = 0.05) indicating that subjective stress ratings increased in the stress group (*F*_(2.566,146.254)_ = 7.60, *p *<* *0.001, η_p_^2^ = 0.12) but not in the control group (*F*_(2.138,130.440)_ = 0.46, *p *=* *0.643, η_p_^2^ = 0.01). As shown in Figure 3, increased subjective stress levels were observed in the stress (vs control) group only after the second encoding block (*t*_(119)_ = 2.84, *p *=* *0.005, *d *=* *0.52), during which the stress manipulation took place, whereas there were no group differences at baseline or in the subsequent blocks (all *p *>* *0.505). Autonomic nervous system activity was measured using EDA, heart rate and blood pressure. For EDA, we observed, again, a significant time × group interaction (*F*_(3.960,451.422)_ = 7.598, *p *<* *0.001, η_p_^2^ = 0.06) showing that EDA levels increased in particular in the stress group (*F*_(3.135,172.451)_ = 9.525, *p *<* *0.001, η_p_^2^ = 0.15). As with the subjective stress ratings, the stress-induced EDA increase was confined to the second encoding block (*t*_(118)_ = 3.427, *p *=* *0.001, *d *=* *0.63), whereas there were no differences at baseline or in any of the subsequent encoding blocks (all *p *>* *0.225). Although there was substantial variability in participants’ heart rate and blood pressure responses to the treatment, we did not obtain a significant effect of the stress manipulation on these parameters at the group level (all *F *<* *1.015, all *p *>* *0.400, all η_p_^2^ < 0.01; and also no significant change from block 1 to block 2, *F*_(1,117)_ = 1.80, *p *=* *0.182, η_p_^2^ = 0.015), suggesting that cardiovascular parameters were overall less sensitive to the stress manipulation (see Table 1).

**Table 1:** Autonomic measures

	Block		1	2	3	4	5	6	7	8	9	10
Control	Syst BP	M (SD)	123.24 (22.50)	118.42 (20.88)	115.93 (21.01)	114.90 (21.89)	115.61 (19.96)	119.33 (18.42)	122.08 (19.00)	120.41 (20.34)	121.43 (21.39)	119.79 (21.54)
	Diast BP	M (SD)	80.31 (17.74)	77.74 (11.11)	79.16 (13.33)	78.36 (11.89)	79.39 (11.33)	81.52 (9.88)	82.81 (10.56)	81.06 (11.34)	83.05 (15.34)	82.00 (12.18)
	HR	M (SD)	86.15 (16.84)	86.79 (18.83)	87.56 (18.96)	88.13 (17.81)	84.08 (16.69)	82.83 (16.03)	80.68 (19.86)	83.90 (18.00)	87.87 (26.79)	90.02 (26.55)
	EDA	M (SD)	2.88 (2.48)	3.01 (2.58)	2.80 (2.68)	2.53 (2.36)	2.83 (2.78)	2.67 (2.37)	3.17 (2.62)	2.84 (2.56)	3.38 (2.73)	2.98 (2.79)
												
Stress	Syst BP	M (SD)	125.21 (15.44)	117.21 (23.58)	119.59 (21.28)	119.97 (19.34)	120.21 (20.96)	120.68 (20.80)	124.04 (18.18)	121.23 (19.51)	121.91 (18.78)	118.72 (21.98)
	Diast BP	M (SD)	82.97 (12.19)	81.14 (15.85)	81.01 (14.24)	81.56 (14.03)	83.33 (14.46)	82.43 (14.81)	84.73 (12.89)	83.05 (13.32)	82.63 (14.35)	80.37 (13.53)
	HR	M (SD)	87.03 (24.48)	89.46 (21.92)	89.25 (24.40)	87.74 (23.18)	85.77 (20.08)	85.78 (17.14)	87.45 (25.42)	90.10 (35.94)	89.34 (45.11)	86.13 (22.89)
	EDA	M (SD)	3.35 (3.12)	5.45 (4.87)	4.26 (4.05)	3.52 (3.93)	3.40 (3.53)	3.10 (3.29)	3.28 (3.35)	3.27 (3.37)	3.65 (3.81)	3.24 (3.48)

Systolic (syst) and diastolic (diast) blood pressure (BP), heart rate (HR) as well as electrodermal activity (EDA) were continuously measured throughout the experiment. Depicted here are the means (M) and SDs of each block.

### Stress decreases prefrontal but increases inferior temporal activity during encoding

**Figure 4.**
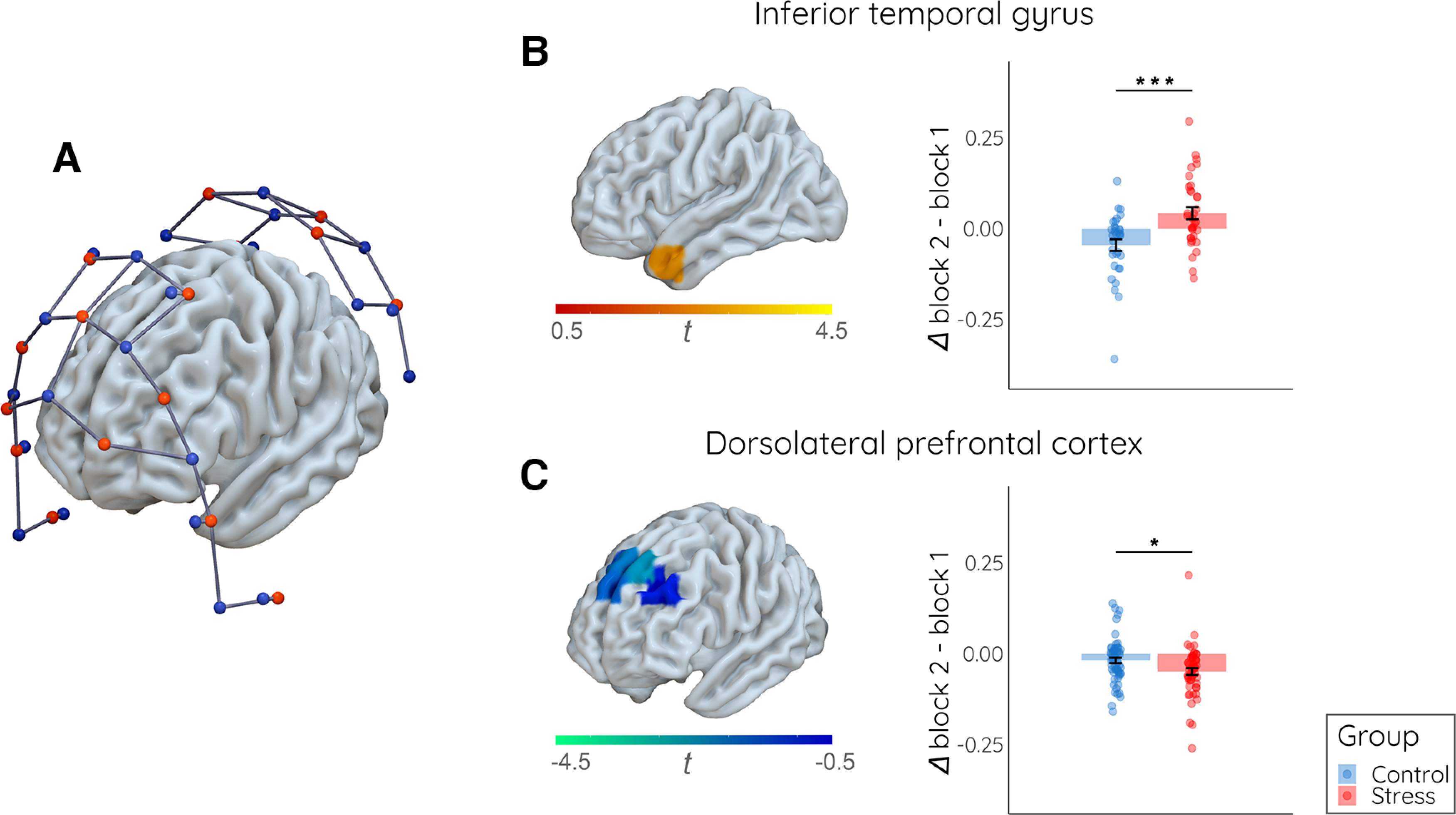
Impact of stress on cortical activity during encoding. ***A***, The fNIRS montage (sources: red; detectors: blue) covered areas of the executive control and salience networks. ***B***, Increased inferior temporal activity (Δ block 2 – block 1) in response to the stress (vs control) manipulation. ***C***, Significant decrease in dlPFC activity (Δ block 2 – block 1) during the stress (vs control) manipulation. Data represent means (±SE); *p < 0.05, ***p < 0.001.

To gain insight into the neural mechanisms associated with potential stress effects on the mode of memory formation, we measured cortical activity during encoding by means of fNIRS. These neural measurements focused on areas of the executive control and salience networks (Fig. 4*A*), including the ITG and dlPFC, that have been implicated in stress and mnemonic processing before (Murray and Ranganath, 2007; Qin et al., 2009; Hermans et al., 2014; Kalbe et al., 2020). We first analyzed which of these areas showed a stress-induced change in activity in the second (i.e., treatment) block of the encoding task (vs the first block as baseline). This analysis revealed significantly increased activity in the ITG during the stress (vs control) manipulation (*t*_(64)_ = 3.805, *p*_corr_ < 0.001, *d *=* *0.94; Fig. 4*B*). In sharp contrast to ITG activity, dlPFC activity decreased significantly in response to the stress manipulation (*t*_(113)_ = 2.562, *p*_corr_ = 0.024, *d *=* *0.48; Fig. 4*C*). Same as for the subjective and autonomic changes, these stress-induced changes in cortical activity where confined to the second task block (all other blocks: all *p*_corr_ > 0.178). As expected, we did not find significant group differences in premotor or somatosensory control areas (all *p *>* *0.140).

### Stress increases memory for individual items but abolishes the recognition priming effect

One day after encoding, participants returned to the lab for a surprise recognition test. Because both sleep (Diekelmann and Born, 2010) and rehearsal (Karpicke and Roediger, 2008) are known to affect memory performance, we asked participants to report their sleep quality and duration for the night between experimental days, as well as the amount of rehearsal of the experimental stimuli or procedures at the beginning of the test day. The stress and control groups did not differ in any of these measures (all *p *>* *0.370; see Table 2). Moreover, groups did not differ in subjective stress ratings, blood pressure or heart rate before the recognition test (all *p *>* *0.155; see Table 3), thus ruling out potential influences of stress at the time of the recognition test.

**Table 2:** Sleep quality and rehearsal between experimental days

	Stress		Control		
Measure	M/N	SD/%	M/N	SD/%	*p*
Unsual sleep	4	6.67	4	6.45	0.966
h/night	7.58	1.18	7.49	0.77	0.647
h last night	7.35	1.24	7.33	0.91	0.922
Insomnia in general	3	5.00	6	9.68	0.492
Insomnia last days	4	6.67	3	4.83	0.715
Thought about d1	36	60	41	66.13	0.574
How many thoughts	1.81	1.95	1.82	1.88	0.979
Talked about d1	35	58.33	31	50.00	0.370
How much bothered	1.33	0.57	1.39	0.78	0.663

After arrival at day 2, participants completed short questionnaires on their sleep quality and the amount of rehearsal after the encoding task on day 1 (d1). No significant difference between the groups were observed on these measures. Data represent means (±SD).

**Table 3:** Stress measures on day 2

	Stress		Control		
Measure	M	SD	M	SD	*p*
Stress rating	18.98	19.75	20.56	23.98	0.694
Syst BP	130.44	25.89	130.94	17.94	0.902
Diast BP	72.93	9.59	70.65	7.79	0.155
HR	75.36	12.66	75.99	12.47	0.782

After arrival at day 2, participants completed a stress rating and their heart rate (HR) and systolic (syst) and diastolic (diast) blood pressure (BP) was measured. No significant difference between the groups were observed on these measures before the memory test. Data represent means (±SD).

Critically, in this recognition test, the sequence of the items was manipulated in a manner that allowed us to probe memory for isolated events as well as recognition priming effects, separately for items encoded in each of the ten encoding blocks. Specifically, an algorithm allocated items that were presented during encoding to specific positions in the recognition test sequence to allow for distinction of three test item categories (in addition to entirely new items that served as lures): (1) old items that followed a new item (single); (2) old items that followed another old item which was, however, presented at least 10 items apart during encoding (remote), thus making recognition priming effects unlikely (Schwartz et al., 2005); and (3) old items that followed an item that preceded this item also during encoding (adjacent) and could thus serve as cue leading to a recognition priming effect (Schwartz et al., 2005; Fig. 1). If memory formation under stress results in enhanced memory formation for individual items but impaired integration of events, then acute stress should enhance subsequent memory for single items but impair memory for adjacent items, compared with a nonstressful control manipulation.

**Figure 5.**
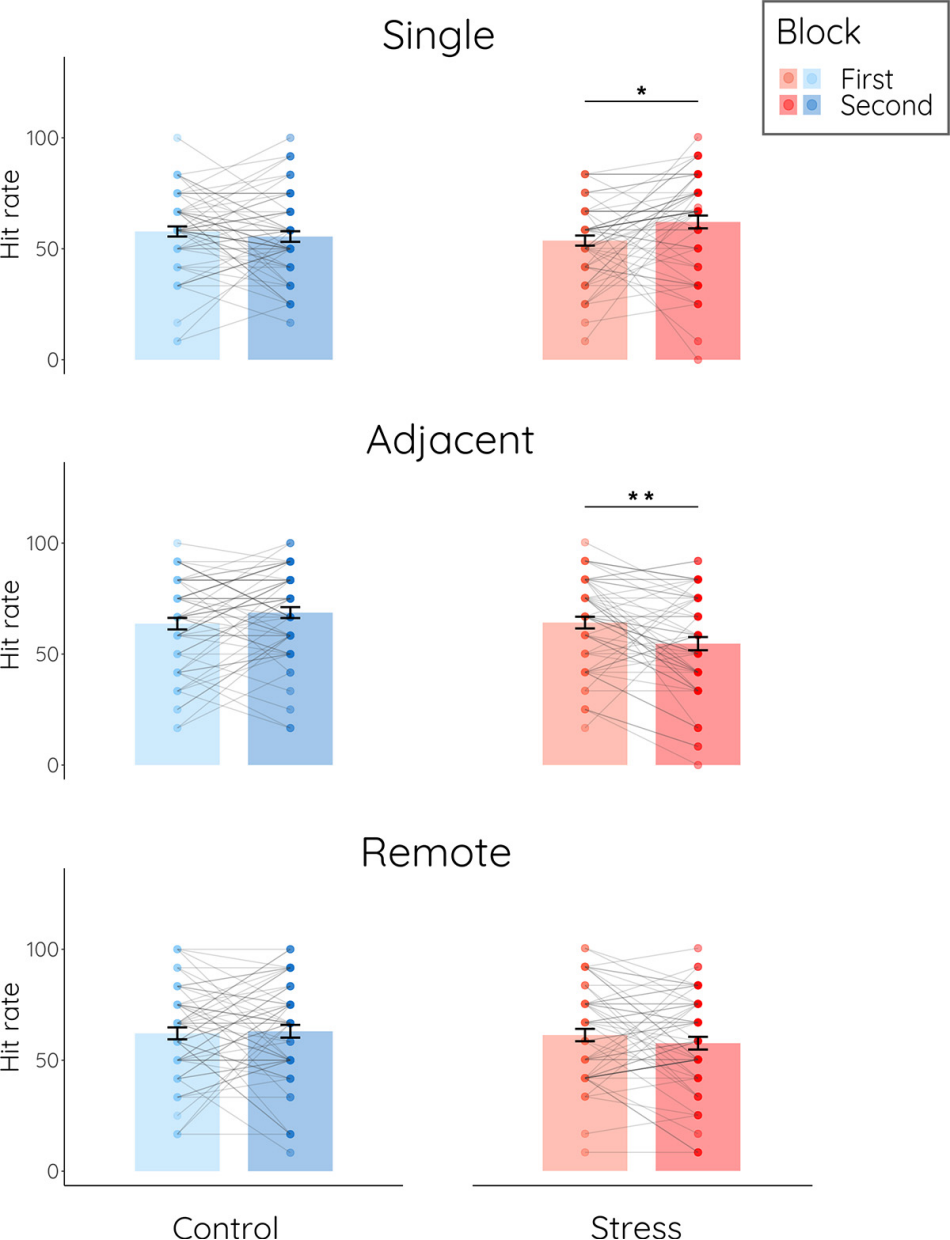
Memory performance on day 2. Hit rates for the first and second block grouped into single, adjacent, and remote items for the control group and stress group. Data represent means (±SE); *p < 0.05, **p < 0.01.

Overall, recognition performance showed the expected recognition priming effect: across groups and blocks, adjacent items (M = 64.07, SEM = 1.36) were significantly better recognized than single items (M = 59.27, SEM = 1.28; *F*_(1,120)_ = 67.079, *p *< 0.001, η_p_^2^ = 0.36); the performance for remote items (M = 62.54, SEM = 1.32) was higher than for single items (*F*_(1,121)_ = 40.366, *p *< 0.001, η_p_^2^ = 0.25) but lower than for adjacent items (*F*_(1,120)_ = 10.236, *p* = 0.002, η_p_^2^ = 0.08; main effect item type: *F*_(1.890,226.874)_ = 43.048, *p *< 0.001, η_p_^2^ = 0.26). The overall false alarm (M = 33.42%, SD = 16.13%) was significantly lower than the overall hit rate (M = 61.95%, SD = 14.10%, *t*_(120)_ = 18.51, *p *< 0.001, *d* = 1.88) and did not differ between groups (*t*_(119)_ = 0.465, *p* = 0.643, *d* = 0.08). In order to probe stress effects on memory for individual items and the recognition priming effect, which is indicative of memory for the association between items (i.e., integrative encoding), we first focused on items from the first (i.e., baseline) and second (i.e., treatment) block of encoding because both the subjective and the autonomic responses to stress were confined to the second encoding block. An item type (single vs remote vs adjacent) × block (first vs second) × group (stress vs control) ANOVA showed a significant three-way interaction (*F*_(1.878,223.426)_ = 12.106, *p *< 0.001, η_p_^2^ = 0.09; Fig. 5). Follow-up tests revealed opposite effects of stress on subsequent recognition of single and adjacent items. For single items, there was a stress-related increase in recognition performance (*F*_(1,119)_ = 6.461, *p* = 0.012, η_p_^2^ = 0.05; Fig. 5). Participants in the stress group showed significantly better memory for single items encoded in block 2 (i.e., under stress) than for those encoded in the baseline block 1 (*t*_(58)_ = 2.558, *p*_corr_ = 0.013, *d* = 0.42), whereas there was no such change in memory in control participants (*t*_(61)_ = 0.862, *p*_corr_ = 0.784, *d* = 0.12). For adjacent items, however, there was a stress-related decrease in memory performance (*F*_(1,119)_ = 14.713, *p *< 0.001, η_p_^2^ = 0.11; Fig. 5). The stress group showed significantly lower memory for adjacent items encoded in block 2 (i.e., under stress) than for adjacent items encoded in block 1 (*t*_(58)_ = 3.343, *p*_corr_ = 0.002, *d* = 0.44), while there was no such difference (but even a trend in the opposite direction) in the control group (*t*_(61)_ = 1.994, *p*_corr_ = 0.102, *d* = 0.25). Accordingly, stressed participants showed relative to controls a significant increase in memory for single items encoded in block 2 compared with those encoded in block 1 (*t*_(119)_ = −2.542, *p*_corr_ = 0.024, *d* = 0.46) but a significant decrease in memory for adjacent items encoded in block 2 compared with those encoded in block 1 (*t*_(119)_ = 3.836, *p*_corr_ < 0.001, *d* = 0.70). Strikingly, acute stress completely abolished the recognition priming effect. While participants in the stress group remembered adjacent items encoded in block 1 significantly better than corresponding single items (*t*_(58)_ = 4.133, *p*_corr_ < 0.001, *d* = 0.55), demonstrating a recognition priming effect for items encoded in the baseline block 1, there was no longer enhanced recognition performance for adjacent relative to single items encoded under stress (item type × block: *F*_(1,58)_ = 18.002, *p*_corr_ < 0.001, η_p_^2^ = 0.24). For items encoded under stress, there tended to be even a reversed recognition priming effect, with better performance for single than for adjacent items (i.e., in block 2; *t*_(58)_ = 2.186, *p*_corr_ = 0.066, *d* = 0.33;). This nonsignificant trend needs to be interpreted with caution though. Compared with nonstressed control participants, stressed participants tended to show enhanced memory for single items (*t*_(119)_ = 1.721, *p* = 0.088, *d* = 0.31) but significantly impaired memory for adjacent items (*t*_(119)_ = 2.113, *p *< 0.001, *d* = 0.67) that were encoded in block 2 (i.e., during the stress manipulation). For remote items, there was no significant influence of stress (*F*_(1,119)_ = 1.136, *p* = 0.289, η_p_^2^ = 0.01). Furthermore, the false alarm rate did not differ across blocks or between groups (all main or interaction effects: all *p* > 0.134, all η_p_^2^ < 0.02).

**Figure 6.**
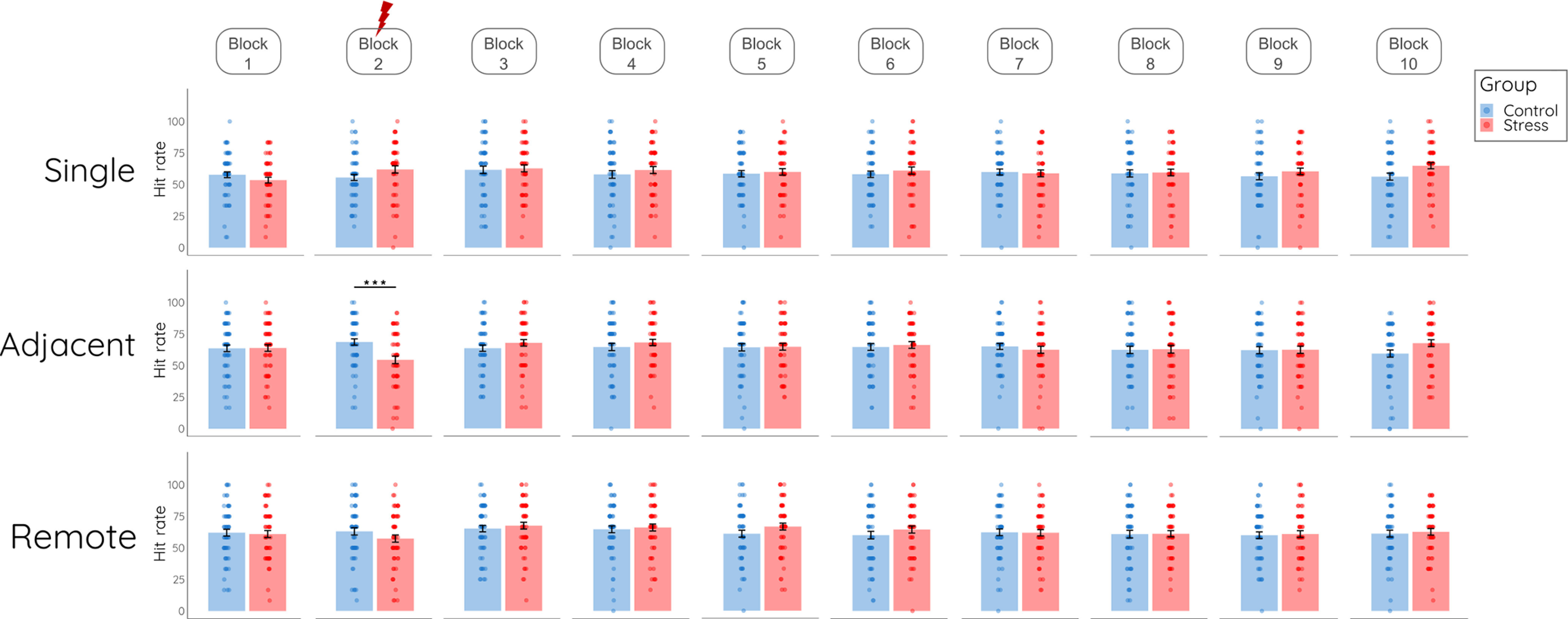
Memory performance on day 2 per item type and block. Hit rates for each block grouped into single, adjacent, and remote items for the control group and stress group. Data represent means (±SE); ***p < 0.001.

In a next step, we extended this analysis to test for potential changes in memory formation for items encoded after the treatment, as indicated in our preregistration. Here, we included all ten blocks in the analysis. In addition to the expected main effect of item type (*F*_(1.901,226.247)_ = 42.926, *p *< 0.001, η_p_^2^ = 0.27) and a main effect of block (*F*_(7.885,938.320)_ = 2.013, *p* = 0.043, η_p_^2^ = 0.02), this analysis showed a significant three-way interaction of item type, block, and group (*F*_(14.963,1780.564)_ = 2.604, *p* = 0.001, η_p_^2^ = 0.02). Follow-up tests revealed that the groups differed only in their memory for single and adjacent items encoded during the treatment block (group × item: *F*_(1,119)_ = 22.137, *p *< 0.001, η_p_^2^ = 0.16) but neither for single and adjacent items encoded in the baseline block (*F*_(1,119)_ = 1.693, *p* = 0.196, η_p_^2^ = 0.01) nor following treatment (all *p* > 0.07; see Fig. 6). When we constrained this analysis to the last eight blocks only, hence excluding the treatment block, the pattern for the last trials remained: there was a main effect of item type (*F*_(2,238)_ = 32.81, *p *< 0.001, η_p_^2^ = 0.22) but no effect of stress (all main or interaction effects including the factor group: all *p* > 0.382, all η_p_^2^ < 0.01), again suggesting that the stress effect was restricted to those items that were encoded under stress.

While the previous analyses collapsed across high confident and low confident hits (in line with previous studies; Henson et al., 2000; Dunsmoor et al., 2015; Elliott et al., 2020), we performed an additional, explorative analysis on high confidence hits only to assess whether stress modulated participants’ confidence in memory (see Schwartz et al., 2005; Gonzalez et al., 2015; Gagnon et al., 2019). Interestingly, this analysis showed that across all blocks the percentage of high confidence hits was higher for adjacent (M = 35.68, SEM = 1.50) than for remote items (M = 34.02, SEM = 1.43) and for remote than for single items (M = 32.14, SEM = 1.30; main effect item: *F*_(2,238)_ = 20.183, *p *<* *0.001, η_p_^2^ = 0.15), but no influence of stress (all main and interaction effects including the factor group: all *p* >* *0.205, all η_p_^2^ < 0.02), suggesting that while memory was reduced for adjacent items and increased for single items encoded under stress, stress seemed to not modulate participants’ subsequent confidence in memory.

After the recognition memory test, participants completed a sequence memory test that was supposed to provide a measure of participants’ explicit memory for the sequence of items during encoding. Specifically, participants were presented with two items that had been presented on day 1 and were requested to first indicate whether these two items had been presented in the same encoding block. If participants indicated that both items were presented in the same block, they were further asked to indicate how many items were presented between these items (ranging from 0–34 items). In retrospect, this task was extremely difficult for participants and their performance was accordingly very low. The accuracy (i.e., hit rate minus false alarm rate) for the response whether items were presented in the same block or not was not above chance level (M = 1.06, SD = 6.09) and in the follow-up rating, the average deviation between the actual distance between items and the distance indicated by participants was 9.08 (SD: 2.73) items across blocks (without differences between blocks: *p* =* *0.956). Groups did not differ in their accuracy in this sequence test (main effect group and group × block interaction for accuracy: both *p *>* *0.359; for average deviation from actual distance: both *p *>* *0.501), which was most likely because of the near floor performance in this test. The discrepancy between the significant recognition priming effect and the low explicit knowledge about item sequence is remarkable as it shows that the item sequence during encoding may serve as a cue that boosts recognition performance (unless participants are stressed), even without explicit knowledge about that sequence.

### Impairment of recognition priming is linked to autonomic arousal and distinct changes in prefrontal and inferior temporal activity

**Figure 7.**
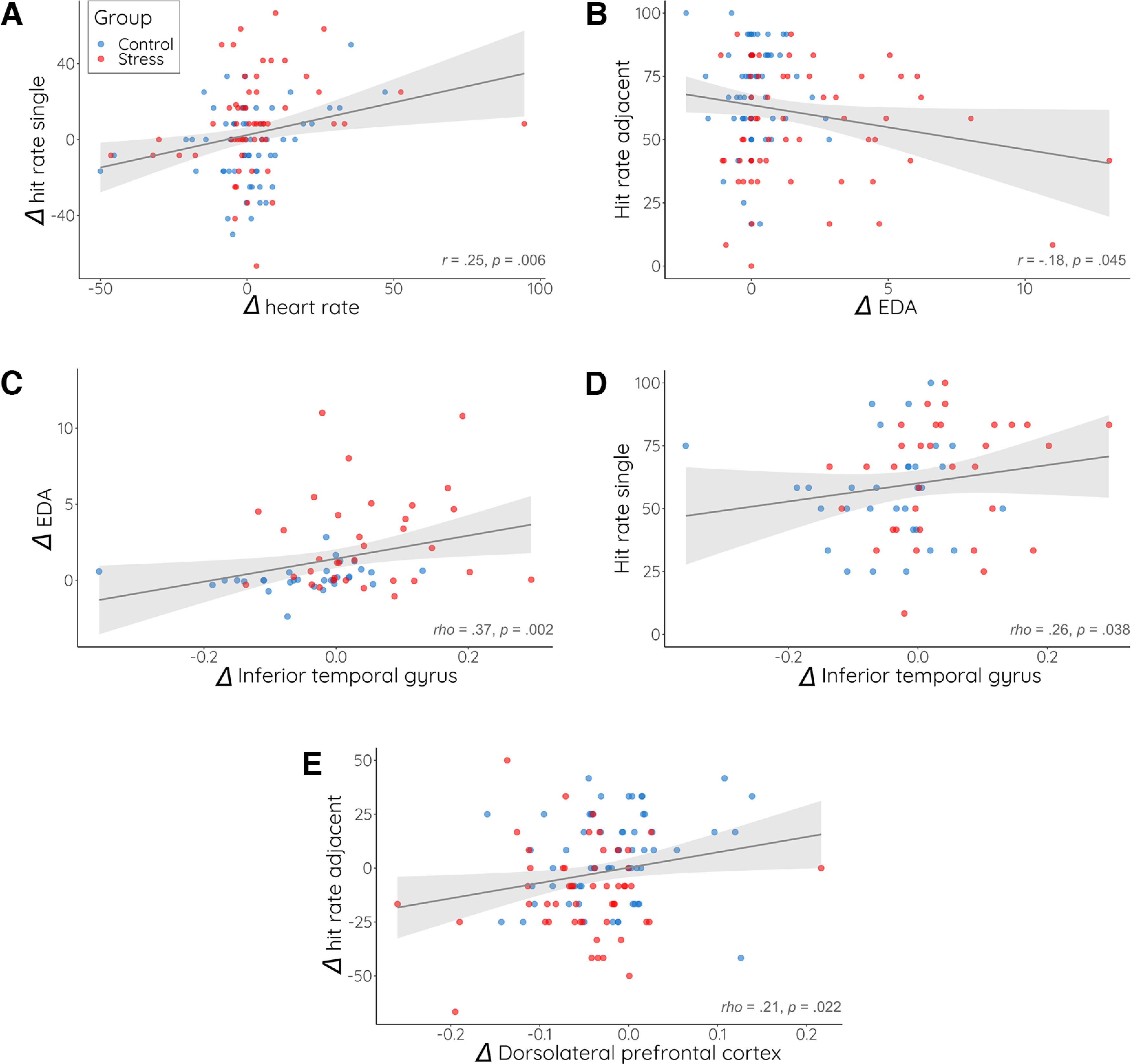
Correlations between memory performance, cortical activity, and autonomic arousal. ***A***, Significant positive correlation between increase in heart rate (bpm) and increase in hits for single items from blocks 1–2. ***B***, Significant negative correlation between electrodermal activity (EDA) increase and hits for adjacent items that were encoded during the treatment (block 2). ***C***, Significant positive correlation between increase in EDA and increase in inferior temporal activity from block 1 to block 2. ***D***, Significant positive correlation between increase in inferior temporal activity and hit rate for single items that were encoded during the treatment. ***E***, Significant positive correlation between the increase in dorsolateral prefrontal (dlPFC) activity and the increase in the hit rate for items encoded during block 2 relative to those encoded during block 1. Each point represents one participant. Fitted regression line with shaded 95% confidence interval. Please see also Extended Data Figures 7-1 and 7-2 for an overview of potentially relevant correlations.

To elucidate the mechanisms through which acute stress enhanced memory for single items but abolished the benefit from the links between items, we first correlated the memory for single and adjacent items with the individual measures of autonomic activity. These analyses showed that the change in memory for single items encoded in treatment block 2 (relative to those encoded in the baseline block 1) was significantly positively correlated with the increase in autonomic activity, expressed as heart rate, from block 1 to block 2 (whole group: *r *=* *0.256, *p* =* *0.006; Fig. 7*A*; control group: *r *=* *0.296, *p* =* *0.023; stress group: *r *=* *0.215, *p* =* *0.104; stress vs control: *z* = 0.457, *p* =* *0.324). Conversely, the memory for adjacent items encoded in the treatment block was negatively correlated with the increase in autonomic activity (expressed as change in EDA; whole group: *r* = −0.187, *p* =* *0.040; Fig. 7*B*; control group: *r* = −0.070, *p* =* *0.591; stress group: *r* = −0.080, *p *=* *0.546; stress vs control: *z* = 0.054, *p *=* *0.479). These correlations suggest that increases in autonomic activity during encoding were associated with both the increased memory for single items and the decreased memory for adjacent items encoded in the treatment block. However, follow-up regression analyses including the factor group as well as the interaction of group and the respective arousal parameter showed that these associations disappeared, when the factor group was included (see Table 4), suggesting, largely in line with the correlations within groups, that the associations between memory and autonomic arousal were largely driven by group differences in arousal.

**Table 4:** Regression analysis

Predictors	Estimates	*t* /*F* value	*p*
Model: single item memory predicted by increase in heart rate			
(Intercept)		−1.99	0.049
Group	0.22	2.49	0.014
Δ heart rate	0.36	1.20	0.234
Group × Δ heart rate	−0.13	−0.42	0.674
*R*^2^/adjusted *R*^2^	0.11/0.09	4.84	0.003
Model: adjacent item memory predicted by increase in EDA			
(Intercept)		12.85	<0.001
Group	−0.30	−3.06	0.003
Δ EDA	−0.23	−0.38	0.703
Group × Δ EDA	0.16	0.26	0.793
*R*^2^/adjusted *R*^2^	0.11/0.09	4.72	0.004
Model: increase in ITG activity predicted by increase in EDA			
(Intercept)		−3.21	0.002
Group	0.39	2.98	0.004
Δ EDA	0.80	0.84	0.406
Group × Δ EDA	−0.69	−0.71	0.481
*R*^2^/adjusted *R*^2^	0.20/0.17	5.29	0.003
Model: single item memory predicted by increase in ITG activity			
(Intercept)		5.76	<0.001
Group	0.09	0.64	0.526
Δ ITG	−0.23	−0.53	0.600
Group × Δ ITG	0.38	0.88	0.381
*R*^2^/adjusted *R*^2^	0.05/0.00	1.01	0.397
Model: adjacent item memory predicted by increase in dlPFC activity			
(Intercept)		12.56	<0.001
Group	−0.29	−2.81	0.006
Δ dlPFC	0.15	0.50	0.619
Group × Δ dlPFC	−0.02	−0.08	0.940
*R*^2^/adjusted *R*^2^	0.12/0.09	4.88	0.003

For predictors, estimates indicate standardized β coefficients. *t* values are shown for predictors, *F* values for *R*^2^/adjusted *R*^2^.

Since our fNIRS data revealed that stress led to increased ITG but decreased dlPFC activity during encoding, we analyzed in a next step whether these opposite changes in cortical activity during encoding were linked to autonomic activity and, more importantly, subsequent memory performance. Correlational analyses showed that increases in ITG activity were, across groups, significantly correlated with an increase in autonomic activity (EDA; whole group: ρ* *= 0.371, *p *=* *0.002; Fig. 7*C*; control group: ρ* *=* *0.433, *p *=* *0.013; stress group: ρ* *= 0.160, *p *=* *0.367; stress vs control: *z* = 1.170, *p *=* *0.121). Even more interestingly, an increase in ITG activity was, across groups, directly associated with the subsequent memory for single items encoded during the treatment block (whole group: ρ* *= 0.258, *p *=* *0.038; Fig. 7*D*; control group: *r *=* *0.134, *p *=* *0.463; stress group: *r *=* *0.307, *p *=* *0.082; stress vs control: z = −0.700, *p *=* *0.242); for remote and adjacent items there were no correlations with the increase in ITG activity though (all correlations within and across groups: all ρ < 0.229, all *p *>* *0.201). While ITG activity appeared to be linked to memory performance for single items, dlPFC activity during the treatment block was significantly correlated with subsequent memory for adjacent items from the treatment block (whole group: ρ* *= 0.21, *p *=* *0.022; Fig. 7*E*; control group: *r *=* *0.279, *p *=* *0.028; stress group: *r* = −0.011, *p *=* *0.937; stress vs control: *z* = 1.535, *p *=* *0.069). Again, we ran follow-up regression analyses including the factor group as well as the interaction between group and the respective predictor. These analyses showed that the associations between ITG activity and EDA as well as the association between memory for adjacent items and dlPFC activity were mainly driven by group differences in EDA and dlPFC activity changes, respectively (see Table 4). The association between ITG activity and single item memory was not supported by the regression analysis.

10.1523/ENEURO.0178-23.2023.f7-1 Extended Data Figure 7-1 Potential correlations between indicators of autonomic arousal and memory performance for single and adjacent items. Data show Pearson correlation coefficients. HR, heart rate; sBP, systolic blood pressure; dBP, diastolic blood pressure; EDA, electrodermal activity; adj, memory for adjacent items; rem, memory for remote items; sing, memory for single items; b2, encoding block 2; Δ, difference encoding block 2 minus encoding block 1. Bold, *p* < 0.050. Download Figure 7-1, DOCX file.

10.1523/ENEURO.0178-23.2023.f7-2 Extended Data Figure 7-2 Potential correlations between inferior temporal gyrus (ITG) or dorsolateral prefrontal cortex (dlPFC) activity and memory performance for single and adjacent items. Data show Spearman rho. Adj, memory for adjacent items; rem, memory for remote items; sing, memory for single items; b2, encoding block 2; Δ, difference encoding block 2 minus encoding block 1. Bold, *p* < 0.050. Download Figure 7-2, DOCX file.

Moreover, it should be noted, for the correlations reported here, that there is, of course, a relatively large number of correlations that could be performed (see also Extended Data Figs. 7-1 and 7-2 for an overview of all correlations) and most of the correlations reported above would not survive a Bonferroni correction for the number of possible correlations. Thus, these correlations must be interpreted with caution.

We further ran mediation analyses testing whether the effects of stress on single and adjacent item memory were mediated by changes in either autonomic arousal (heart rate, blood pressure, or EDA) or cortical (i.e., ITG or dlPFC) activity. These analyses revealed a trend for a mediation of stress effects on the change in memory for single items from block 2 relative to those encode in block 1 by changes in EDA from baseline to treatment (indirect effect: β = −3.482, *p *=* *0.083, 95% CI: −7.421–0.457). Apart from this nonsignificant trend, none of the other mediation models approached statistical significance (indirect effects: all β < 3.42, all *p *>* *0.164), suggesting that the observed stress effects on single item and adjacent item memory were not directly mediated by stress-induced changes in autonomic arousal or cortical activity.

### Control variables

We measured participants’ subjective chronic stress levels, depressive mood, and state as well as trait anxiety as control variables and show that the groups did not differ on any of these measures (all *p *>* *0.426; see Table 5).

**Table 5:** Control variables

	Stress		Control		
Measure	M	SD	M	SD	*p*
STAI-T	39.27	10.77	38.05	10.48	0.533
STAI-S D1	34.45	7.33	35.50	8.04	0.455
STAI-S D2	34.63	8.78	34.10	8.10	0.727
TICS	14.57	9.09	13.76	9.60	0.640
BDI	7.15	7.22	6.18	5.83	0.426

After arrival at day 1, participants completed the questionnaires STAI-T, STAI-S D1, TICS, BDI, and STAI-S D2 was completed at the beginning of the experiment on day 2. No significant difference between the groups were observed on these measures. Data represent means (±SD).

## Discussion

Stress has a major impact on memory, with important implications for educational contexts and stress-related mental disorders, such as phobia or PTSD (Parsons and Ressler, 2013; Vogel and Schwabe, 2016a; de Quervain et al., 2017). These effects of stress on memory, however, may be more complex than commonly assumed and not all aspects of a stressful episode may be remembered equally well. In this preregistered study, we tested a mode of memory formation under stress that promotes subsequent memory for individual elements of an episode but processes these elements separately, resulting in isolated and disintegrated memories (for similar proposals of enhanced item but impaired associative memory under high arousal, see Mather, 2007; Bisby and Burgess, 2017). In line with this proposed mechanism, our data indicated that participants showed enhanced memory for individual items encoded under stress but a reduced capacity to benefit from the cueing by stimuli presented in close temporal proximity to the target items during encoding, suggesting that the elements of the stressful episode were less well linked to one another. These changes in memory formation were, across groups, linked to autonomic arousal, with memory for individual items being positively correlated with heart rate increases and temporal sequence effects in memory being negatively correlated with increases in EDA. Furthermore, these changes in memory formation were, again across groups, accompanied by opposite changes in dlPFC and ITG activity. Notably, however, these associations were mainly driven by differences between groups in the parameters. Moreover, mediation analyses showed that neither these opposite changes in dlPFC and ITG activity after stress, nor stress-induced changes in autonomic activity, directly mediated the observed impact of stress on memory.

A central tenet of memory research holds that our ability to remember an event depends on the availability of relevant encoding-related cues at recall (Godden and Baddeley, 1975; Eich, 1980). In agreement with this assumption, the recognition priming phenomenon shows that items that preceded a target item during encoding may foster later recognition of this target, when presented immediately before the target item during the recognition test (Schwartz et al., 2005). We show here that stress during encoding abolishes this recognition priming effect. As there was a 24-h interval between encoding and test and groups did not differ in stress levels before the memory test, we assume that stress affected mnemonic processes at encoding and not during the recognition test. We propose that stress disrupted the encoding of associations between events as well as their temporal sequence, thereby reducing the capacity of individual events to cue the memory of other events at test. Interestingly, while this reduction in temporal sequence memory was observed in the recognition test performance, explicit sequence memory was overall very low and not modulated by stress.

An alternative interpretation of the observed stress effect could be that stress did not selectively affect the association between events, but rather acted as a distractor that affected memory for the events encoded under stress per se, resulting in overall weaker memories that thus could not serve as cues to support recognition. However, this interpretation is refuted by the finding that memory for single items encoded under stress was enhanced, not impaired. This enhanced memory for individual items may have been because of increased attentional processing and the prioritization of stressor-related cues, linked to a stress-induced bias toward the salience network (Hermans et al., 2014), and is generally in line with the previously reported memory enhancement for central elements of a stressful episode (Kalbe et al., 2020). Thus, compared with a nonstressful control condition, in which associative cueing mechanisms boosted memory for adjacent items, stress exerted opposite effects on memory for single and adjacent items. Whereas stress reduced the associative cueing potential of events to facilitate memory of other encoded events, presumably by impairing the integrative encoding of events, it enhanced single item memory, thus making the memory for adjacent and single items comparable and abolishing the recognition priming effect. Remarkably, there was even a nonsignificant trend for better memory for single items than for adjacent items encoded under stress (i.e., a reversed recognition priming effect). Yet, this (rather surprising) trend needs to be interpreted with caution.

Importantly, both the increased memory for single items and the reduced memory for adjacent items were, across groups, linked to autonomic arousal. This finding dovetails with evidence that the stress-induced reconfiguration of large-scale neural networks toward the salience network is mainly driven by noradrenergic arousal (Hermans et al., 2011). As expected, autonomic arousal vanished relatively quickly once the stressful encounter was over. Accordingly, the observed changes in memory were limited to the events encoded under stress. However, it should be noted that we tested healthy participants and that patients or individuals at increased (e.g., familial) risk for stress-related mental disorders might exhibit longer-lasting stress responses that result in less circumscribed alterations in memory. Further, the associations with autonomic arousal were observed across groups and not specifically in stressed participants. Moreover, mediation analyses showed, except for a nonsignificant trend for mediation of stress effects on single item memory by increases in EDA, no evidence for a direct mediation of stress-induced changes in single and adjacent item memory by measures of autonomic arousal and regression analyses suggested that the observed correlations across groups were mainly because of group differences in autonomic arousal. In addition, it has been suggested that delayed stress responses, mediated by genomic glucocorticoid action, may have opposite effects that serve to contextualize or rationalize the stressful episode (Hermans et al., 2014; Schwabe et al., 2022). In the present study, we did not observe any effects for events encoded after the stressful episode. This, however, could be owing to the duration of the encoding session, as genomic glucocorticoid actions develop only 60–90 min poststress (Joëls et al., 2011).

At the neural level, our fNIRS data revealed opposite effects of stress on ITG and dlPFC activity at encoding. The ITG is part of the salience network that is preferentially recruited under stress and thought to prioritize the processing of stressor-related information (Hermans et al., 2014). In line with the proposed stress-induced bias toward the salience network, we obtained here increased ITG activity under stress that was linked to autonomic arousal. Moreover, the increase in ITG activity from baseline to treatment correlated with the subsequent memory for single items encoded during the treatment block. This finding extends studies suggesting a role of the ITG in memory formation under stress (Henckens et al., 2009; Kalbe et al., 2020) by showing that this area facilitates in particular the encoding of individual elements of an episode. In contrast to ITG activity, dlPFC activity was significantly reduced under stress. This result corroborates earlier findings suggesting that stress impairs dlPFC functioning (Arnsten, 2009; Qin et al., 2009) and reconfigures large-scale networks at the cost of the executive control network (Hermans et al., 2014), which includes the dlPFC. Beyond its well-documented role in working memory (Curtis and D’Esposito, 2003), the dlPFC has been assigned a key role in building relationships between elements of an episode during encoding (Murray and Ranganath, 2007). The idea that the dlPFC is critically involved in relational encoding dovetails with our finding that dlPFC activity correlated, across groups, with memory for adjacent items, which is assumed to reflect the degree of integration of events at encoding. Thus, while enhanced ITG activity may have promoted the encoding of individual events under stress, the reduced integration of these events at encoding and consequently the reduced memory for adjacent items may be owing to a stress-induced impairment of dlPFC functioning. At this point, it needs to be noted, however, that, as for autonomic arousal, there seemed to be no direct mediation of the stress effect on memory by altered ITG or dlPFC activity and that the observed associations appeared to be mainly driven group differences in these parameters. More generally, it is important to note that while fNIRS provides valuable information about cortical activity, this technique does not allow the measurement of subcortical activity and is limited to a relatively small set of predefined regions. We assume that the altered mode of memory formation under stress that we have observed here is not mediated by single brain areas but by a network of interconnected regions, including subcortical areas such as the hippocampus, amygdala or dorsal striatum (Hermans et al., 2014; Schwabe et al., 2022).

Finally, some limitations of the present study need to be addressed. First, while we observed significant increases in subjective stress levels and EDA in response to our stressor, we did not observe a similar increase in cardiovascular parameters. Further, cortisol concentrations, which would have been interesting to evaluate potential delayed effects, were not available. Future studies could use more potent stressors, although it remains crucial that the stressor is directly related to the learning experience and it is to be noted that we did observe the predicted changes in memory even using this rather moderate stressor. Second, performance in the explicit sequence memory test was very low. This “floor effect” could be because of the actual lack of explicit sequence memory, which would be an interesting finding, but also to the test not being sensitive enough. Thus, future studies should use more elaborated tests of explicit sequence memory to test for the potential dissociation in explicit and implicit sequence memory. In general, future studies are required to replicate the present pattern of results. In particular, these studies should assess the robustness of the opposite correlations between autonomic arousal and memory for single and adjacent items as these were not consistently observed across different arousal measures and obviously, the number of potential correlations comes with an increased risk of false positives.

In sum, we show here that not all aspects of memory for a stressful episode are strengthened. Instead, our data indicate that encoding under stress results in a memory formation mode that boosts the memory for individual elements of the episode but appears to reduce the processing of the associations between these elements, which resembles memory distortions that have been previously described in stress-related disorders such as PTSD (Brewin, 2011). Together, our findings lend support to recent dual-representation accounts of trauma memory (Bisby et al., 2020) that assume increased sensory representations but reduced hippocampal context representations for emotionally arousing events, which might be reflected in the increased memory for single events but reduced memory for adjacent events seen here. These results might further provide a potential mechanism to explain the strong but fragmented memory in PTSD, and point to potential targets for treating this debilitating disease.

## Synthesis

Reviewing Editor: Frederike Beyer, Queen Mary University of London Decisions are customarily a result of the Reviewing Editor and the peer reviewers coming together and discussing their recommendations until a consensus is reached. When revisions are invited, a fact-based synthesis statement explaining their decision and outlining what is needed to prepare a revision will be listed below. The following reviewer(s) agreed to reveal their identity: NONE. Note: If this manuscript was transferred from JNeurosci and a decision was made to accept the manuscript without peer review, a brief statement to this effect will instead be what is listed below.

Thank you for your responses to the reviewer comments, and the corresponding edits to your manuscript. Most points are addressed satisfactorily, and I do not think further reviews are required. However, I agree with the comment of reviewer 2, that the interpretation of the correlation analyses can be problematic, as it may be driven by group effects. To address this point, please include analyses to directly test this (in a regression including the group factor, does arousal / brain activity remain a significant predictor?), or at least directly discuss this point when interpreting correlation results.

## Author Response

Thank you for your responses to the reviewer comments, and the corresponding edits to your manuscript. Most points are addressed satisfactorily, and I do not think further reviews are required. However, I agree with the comment of reviewer 2, that the interpretation of the correlation analyses can be problematic, as it may be driven by group effects. To address this point, please include analyses to directly test this (in a regression including the group factor, does arousal / brain activity remain a significant predictor?), or at least directly discuss this point when interpreting correlation results.

RESPONSES: As suggested, we now report the full model details for the regression analyses, including the factor group and the respective interaction effect including the factor group. We present these data in the new Table 4. Moreover, we explicitly refer to the findings of this regression analyses on page 21, lines 510 to 514:

“However, follow-up regression analyses including the factor group as well as the interaction of group and the respective arousal parameter showed that these associations disappeared, when the factor group was included (see table 4), suggesting, largely in line with the correlations within groups, that the associations between memory and autonomic arousal were largely driven by group differences in arousal.”

And on page 22, lines 529 to 534:

“Again, we ran follow-up regression analyses including the factor group as well as the interaction between group and the respective predictor. These analyses showed that the associations between ITG activity and EDA as well as the association between memory for adjacent items and dlPFC activity were mainly driven by group differences in EDA and dlPFC activity changes, respectively (see table 4). The association between ITG activity and single item memory was not supported by the regression analysis.”

Furthermore, we address the fact that the regression analyses showed that the observed correlations were mainly due to group differences in the respective parameters explicitly and repeatedly in the discussion. Please see page 23, lines 570/571:

“Notably, however, these associations were mainly driven by differences between groups in the parameters.”

On page 25, lines 616/617:

”...and regression analyses suggested that the observed correlations across groups were mainly due to group differences in autonomic arousal.”

And on page 26, lines 644/645:

”...and that the observed associations appeared to be mainly driven group differences in these parameters.”

Although the regression analyses suggest that the observed correlations between performance, brain activity and autonomic arousal were mainly driven by group differences in these variables, we still think that the reported correlations are potentially interesting and informative for readers and should therefore remain in the manuscript (also for the sake of transparency). However, in light of the findings of the regression analyses, we decided to remove references to the obtained correlations from the abstract and significance statement (as well as from the final paragraph of the discussion), to make these correlations less prominent in the manuscript.
