# Supplementary material for: Strong but Fragmented Memory of a Stressful Episode
Source: eNeuro. 2023 Sep 1;10(9):ENEURO.0178-23.2023. doi: 10.1523/ENEURO.0178-23.2023 (PMC10484358; doi:10.1523/ENEURO.0178-23.2023)
Supplement: Extended Data Figure 7-1 — Potential correlations between indicators of autonomic arousal and memory performance for single and adjacent items. Data show Pearson correlation coefficients. HR, heart rate; sBP, systolic blood pressure; dBP, diastolic blood pressure; EDA, electrodermal activity; adj, memory for adjacent items; rem, memory for remote items; sing, memory for single items; b2, encoding block 2; Δ, difference encoding block 2 minus encoding block 1. Bold, p < 0.050. Download Figure 7-1, DOCX file. [file enu-eN-NWR-0178-23-s01.docx]

|  | Whole group | | | | | | Control group | | | | | | Stress group | | | | | |
| --- | --- | --- | --- | --- | --- | --- | --- | --- | --- | --- | --- | --- | --- | --- | --- | --- | --- | --- |
|  | Adj b2 | Δ adj | Rem b2 | Δ rem | Sing b2 | Δ sing | Adj b2 | Δ adj | Rem b2 | Δ rem | Sing b2 | Δ sing | Adj b2 | Δ adj | Rem b2 | Δ rem | Sing b2 | Δ sing |
| HR bl2 | .060 | .050 | -.002 | -.043 | .046 | .113 | .005 | -.064 | -.104 | -.104 | .165 | .222 | .157 | .196 | .104 | .031 | -.065 | .007 |
| Δ HR | .067 | -.049 | .078 | .035 | **.204** | **.255** | -.062 | .033 | .020 | .245 | .119 | **.296** | .206 | -.076 | .146 | -.150 | .249 | .215 |
| sBP bl2 | .025 | -.036 | .079 | .044 | -.046 | -.106 | .080 | -.078 | .197 | .045 | .199 | .075 | -.034 | -.023 | -.040 | .038 | -.231 | -.235 |
| Δ sBP | .107 | .166 | .006 | .073 | -.102 | -.161 | .190 | .105 | .083 | .006 | .077 | -.095 | -.006 | .206 | -.103 | .148 | -.257 | -.205 |
| dBP bl2 | .021 | -.031 | .028 | .034 | -.077 | -.008 | .009 | -.092 | .138 | -.048 | .216 | .249 | .107 | .081 | -.022 | .009 | **-.295** | -.212 |
| Δ dBP | .065 | .121 | -.097 | -.023 | -.110 | -.133 | .044 | .081 | -.046 | .093 | .039 | -.059 | .113 | .192 | -.140 | .011 | -.241 | -.213 |
| EDA bl 2 | -.085 | -.130 | -.054 | -.121 | .014 | -.070 | -.021 | **-.259** | .075 | -.158 | .247 | .044 | -.035 | .086 | -.137 | -.049 | -.228 | -.246 |
| Δ EDA | **-.187** | -.093 | **-.187** | -.090 | -.084 | -.061 | -.070 | .006 | -.144 | -.202 | .086 | .198 | -.080 | .058 | -.179 | -.013 | -.248 | **-.288** |

**Figure 7-1: Potential correlations between indicators of autonomic arousal and memory performance for single and adjacent items**

Data show Pearson correlation coefficients. HR – heart rate, sBP – systolic blood pressure, dBP – diastolic blood pressure, EDA – electrodermal activity, adj – memory for adjacent items, rem – memory for remote items, sing –memory for single items, b2 – encoding block 2, Δ – difference encoding block 2 minus encoding block 1. **Bold** – *P* < .050
