# Supplementary material for: Strong but Fragmented Memory of a Stressful Episode
Source: eNeuro. 2023 Sep 1;10(9):ENEURO.0178-23.2023. doi: 10.1523/ENEURO.0178-23.2023 (PMC10484358; doi:10.1523/ENEURO.0178-23.2023)
Supplement: Extended Data Figure 7-2 — Potential correlations between inferior temporal gyrus (ITG) or dorsolateral prefrontal cortex (dlPFC) activity and memory performance for single and adjacent items. Data show Spearman rho. Adj, memory for adjacent items; rem, memory for remote items; sing, memory for single items; b2, encoding block 2; Δ, difference encoding block 2 minus encoding block 1. Bold, p < 0.050. Download Figure 7-2, DOCX file. [file enu-eN-NWR-0178-23-s02.docx]

**Figure 7-2: Potential correlations between inferior temporal gyrus (ITG) or dorsolateral prefrontal cortex (dlPFC) activity and memory performance for single and adjacent items**

|  | Whole group | | | | | | Control group | | | | | | Stress group | | | | | |
| --- | --- | --- | --- | --- | --- | --- | --- | --- | --- | --- | --- | --- | --- | --- | --- | --- | --- | --- |
|  | Adj b2 | Δ adj | Rem b2 | Δ rem | Sing b2 | Δ sing | Adj b2 | Δ adj | Rem b2 | Δ rem | Sing b2 | Δ sing | Adj b2 | Δ adj | Rem b2 | Δ rem | Sing l2 | Δ sing |
| Δ ITG | .020 | .028 | .004 | -.130 | **.258** | .188 | .036 | .146 | -.138 | -.139 | .134 | .022 | .288 | .228 | .174 | -.036 | .307 | .250 |
| Δ dlPFC | .137 | **.217** | .078 | **.239** | -.182 | -.065 | .100 | **.279** | .101 | **.363** | .084 | .184 | .015 | .-011 | -.055 | -.031 | **-.313** | -.153 |

Data show Spearman *rho*. Adj – memory for adjacent items, rem – memory for remote items, sing – memory for single items, b2 – encoding block 2, Δ – difference encoding block 2 minus encoding block 1. **Bold** – *P* < .050
